# Comparison of two Norepinephrine rescue bolus for Management of Post-spinal Hypotension during Cesarean Delivery: a randomized controlled trial

**DOI:** 10.1186/s12871-020-01004-y

**Published:** 2020-04-17

**Authors:** Yasmin S. Hassabelnaby, Ahmed M. Hasanin, Nada Adly, Maha M. A. Mostafa, Sherin Refaat, Eman Fouad, Mohamed Elsonbaty, Hazem A. Hussein, Mohamed Mahmoud, Yaser M. Abdelwahab, Ahmed Elsakka, Sarah M. Amin

**Affiliations:** 1 Department of anesthesia and critical care medicine, 01 elsarayah street, Elmanyal, Cairo, 11559 Egypt; 2 Department of anesthesia and critical care medicine, Beni-Suef university, Beni-Suef, Egypt

**Keywords:** Cesarean delivery, Hypotension, Spinal anesthesia, Norepinephrine

## Abstract

**Background:**

Data on the best norepinephrine bolus dose for management of hypotension are limited. The aim of this study was to compare the efficacy and safety of two norepinephrine bolus doses in the rescue management of maternal hypotension during cesarean delivery.

**Methods:**

This randomized, controlled trial included mothers scheduled for cesarean delivery with spinal anesthesia with a prophylactic norepinephrine infusion. Following spinal anaesthesia administration, a participant was considered hypotensive if systolic blood pressure was ≤80% compared to the baseline reading. Participants were allocated to receive either 6 mcg or 10 mcg norepinephrine bolus for the management of hypotensive episodes. The hemodynamic response after administration of norepinephrine bolus was recorded. The episode was considered successfully managed if systolic blood pressure returned to within 80% from the baseline reading within 2 min after norepinephrine bolus administration, and did not drop again within 6 min after the norepinephrine bolus. The primary outcome was the incidence of successful management of the first hypotensive episode. Other outcomes included systolic blood pressure, heart rate, incidence of maternal bradycardia, and reactive hypertension.

**Results:**

One hundred and ten mothers developed hypotensive episodes and received norepinephrine boluses for management. The number of successfully managed first hypotensive episodes was 50/57 (88%) in the 6 mcg-treated episodes and 45/53 (85%) in the 10 mcg-treated episodes (*p* = 0.78). Systolic blood pressure was comparable after administration of either bolus dose. Heart rate was lower after administration of 10 mcg bolus compared to 6 mcg bolus, without significant bradycardia requiring atropine administration. The incidence of reactive hypertension was comparable between both groups.

**Conclusion:**

In mothers undergoing elective cesarean delivery under prophylactic norepinephrine infusion at 0.05 mcg/kg/min, there was no advantage to the use of 10 mcg norepinephrine bolus over 6 mcg norepinephrine bolus for the rescue management of first hypotensive episode. Neither of the 2 bolus doses reached a 100% success rate. The incidences of bradycardia and reactive hypertension were comparable between both norepinephrine doses.

**Trial registration:**

At clinicaltrial.gov registry system on January 4, 2019 Clinical trial identifier: NCT03792906.

## Background

Maternal hypotension after subarachnoid block is a frequent and deleterious complication during cesarean delivery. The latest consensus for management of spinal hypotension during cesarean delivery recommends the use of prophylactic vasopressors in all non-hypertensive mothers [1]; however, maternal hypotension is still present even in mothers receiving prophylactic vasopressors. Thus, management of maternal hypotension using vasopressor boluses is necessary [2]. The commonly used vasopressors during cesarean delivery are ephedrine, phenylephrine, and recently norepinephrine (NE) [2]. The use of ephedrine may be accompanied by maternal tachycardia and neonatal acidosis [2]. Phenylephrine is still the first line medication for prevention and management of maternal hypotension; however, its use might result in bradycardia and decreased maternal cardiac output [2, 3]. Norepinephrine is an alpha adrenergic agonist with weak beta adrenergic agonistic activity; thus, it does not cause maternal bradycardia as frequently as does phenylephrine [4]. Norepinephrine infusion for prophylaxis against maternal hypotension is showing promising results [4–6]. Therefore, NE infusion is being recognized as a good alternative to phenylephrine infusion during cesarean delivery. However, the use of NE boluses for the management of maternal hypotension has not been adequately explored. Few studies have reported the use of NE bolus for the management of hypotension during cesarean delivery. However, the optimum dose for NE bolus in mothers receiving prophylactic NE infusion is unclear. An insufficient NE bolus would lead to failed management and a prolonged hypotensive episode, whereas a higher dose might lead to reactive hypertension and/or bradycardia, which is sometimes severe. Therefore, determining the optimum dose for NE bolus would enable proper control of maternal hemodynamic profile. In this study, we tested the hypothesis that a 10 mcg NE bolus is more effective than a 6 mcg NE bolus for rescue management of the first maternal hypotensive episode after subarachnoid block during cesarean delivery while using a prophylactic norepinephrine infusion.

## Methods

A randomized, double-blinded, controlled trial was conducted in the obstetric theatre, Cairo University Hospital from January 2019 to April 2019. The study was approved by Cairo University research ethics committee (N-71-2018) and was registered before recruitment of the first participant at clinicaltrial.gov registry system on January 4, 2019 (clinical trial identifier: NCT03792906, principal investigator: Ahmed Hasanin). The study adheres to CONSORT guidelines. Prior to enrollment in the study, written informed consent was obtained from the participants. A computer-generated sequence was prepared by the principal investigator through an online random number generator. The generated codes for participants were placed into sequentially numbered opaque envelopes. Each envelope included the instructions for preparing the drug bolus. The envelope was opened by an anesthesia resident (who was not involved in patient management) who was responsible for preparing the study drug. Multiple syringes of the same dose, either 6 mcg or 10 mcg NE diluted in 10 mL, were prepared for each patient before initiation of anesthesia, and delivered to the blinded anesthetist-in-charge who was responsible for administration of the bolus and recording the data. If the participant did not have any hypotensive episode, the randomization code was returned to a new similar envelope to be re-used with the next mother.

Our study included non-laboring mothers, with term, singleton pregnancy, admitted for elective cesarean delivery and aged between 18 and 40 years. Exclusion criteria were peripartum bleeding, coagulation disorders, history of uncontrolled cardiovascular morbidities, and baseline systolic blood pressure (SBP) > 140 mmHg or < 100 mmHg.

Upon arrival to the operating room, participants were monitored using electrocardiography, pulse oximetry, and non-invasive blood pressure monitoring. An 18G cannula was inserted, and pre-medication drugs were delivered (metoclopramide 10 mg and ranitidine 50 mg). Blood pressure was non-invasively monitored using General Electric (GE, Solar™ 8000i) monitor. Three blood pressure readings, with a difference of < 10%, were obtained at 2-min intervals, and their mean was used as the baseline blood pressure reading. Lactated Ringer’s co-load was rapidly initiated at the time of spinal injection at a rate of 15 mL.Kg^− 1^ over 10 min [7]. Eleven milligrams (2.2 mL) hyperbaric bupivacaine plus 20 mcg fentanyl were injected in the L3-L4 or L4-L5 interspace. The spinal block was performed in the sitting position using a 25G spinal needle, and the participant was then positioned supine with a left-lateral uterine tilt. Pinprick was used for evaluation of block success 5 min after intrathecal injection. The block was considered successful if the sensory block level was at least at T4.

After obtaining cerebrospinal fluid, all participating mothers received NE infusion in the same line running with intravenous fluids at a rate of 0.05 mcg. Kg^− 1^.min^− 1^. Norepinephrine was prepared in a 50-mL syringe (8 mcg.mL^− 1^) [5]. Systolic blood pressure was recorded starting from the baseline pre-injection reading at 1-min intervals for 30 min after intrathecal injection, followed by 5-min intervals till the end of the operation. Fluid administration continued up to a maximum of 1.5 l. After delivery, an oxytocin bolus (0.5 IU) was delivered over 5 s, followed by infusion at a rate of 2.5 IU.hr.^− 1^. Norepinephrine infusion was stopped 5 min after delivery.

The participant was considered to have a hypotensive episode when SBP was ≤80% of the baseline reading during the period starting from intrathecal injection of local anesthetic until the delivery of the fetus. A severe hypotensive episode was defined as SBP ≤60% of the baseline reading. A hypotensive episode was managed by injection of an NE bolus, either 6 mcg or 10 mcg, according to the group code. The hypotensive episode was considered as successfully managed if maternal SBP returned to > 80% of the baseline reading within 2 min. The NE bolus was considered a failure if maternal SBP failed to reach the target value within 2 min after the bolus, or if SBP dropped again to < 80% of the baseline reading within 6 min from the NE bolus. In case of a failed bolus, an additional NE bolus with the same dose was administrated. The additional NE bolus, which was given for management of a failed bolus, was not included in the analysis.

If the participant mother developed hypertension (SBP ≥120% from the baseline reading), the NE infusion was paused till the next SBP reading, and then re-started in a reduced rate (50% of the pre-episode rate) when the SBP decreased to within 20% of the baseline reading. Bradycardia (defined as heart rate less than 55 bpm) was treated by stoppage of the vasopressor infusion (if not associated with hypotension). If the bradycardia was associated with hypotension, an atropine bolus (0.5 mg) was administered.

The two groups of mothers, namely the 6 mcg group and the 10 mcg group, were compared with regard to the response to the first rescue NE bolus and the neonatal outcomes.

### Primary outcome

The rate of successful management of the first maternal hypotensive episode.

### Secondary outcomes

The rate of successful management of a severe maternal hypotensive episode, the incidence of reactive hypertension (defined as SBP ≥120% from the baseline reading after administration of the first NE bolus), SBP (baseline reading, pre-episode reading, 1-min, 2-min, 4-min, and 6-min post-episode readings), heart rate (baseline reading, pre-episode reading, 1-min, 2-min, 4-min, and 6-min post-episode readings), incidence of intraoperative nausea “defined as unpleasant sensation which is associated with an awareness of the urge to vomit”, incidence of intraoperative vomiting “defined as forceful expulsion of gastric contents from the mouth” [8], intraoperative requirements of NE, and atropine, time between spinal block and delivery of the fetus, umbilical blood gases (*p*H, PCO_2_, PO_2_, lactate, and HCO_3_), and Apgar score for the fetus at 1 min and 5 min post-delivery.

### Statistical analysis and sample size calculation

Our primary outcome was the rate of successful management of maternal hypotension. As there are no available data for this outcome using NE boluses, we performed a pilot study in which we reported a rate of successful management of maternal hypotension of 77% with the 6 mcg bolus. G-power software (version 3.1.9.2) was used to calculate the sample size. An absolute improvement of 18% in the rate of successful management of a first hypotensive episode (aiming 95% success rate) was planned for sample size calculation. One-hundred and four hypotensive mothers (52 per group) at least were estimated to have a study power of 80% and an alpha error of 0.05. This number was increased to 112 mothers (56 per group) to compensate for possible dropouts.

Analysis of data was performed using Statistical package for social science (SPSS) software, version 15 for Microsoft Windows (SPSS Inc., Chicago, iL, USA). Categorical data were reported as numbers and percentages and were analyzed using chi-squared test. Normality of continuous data was evaluated using Kolmogorov-Smirnov test. Continuous data with normal distribution were presented as means (standard deviations) and were analyzed using unpaired student t-test. Skewed data were presented as medians (quartiles) and were analyzed using Mann Whitney U test. Two-way repeated measures ANOVA was used to evaluate bolus dose (between-groups factor) and time (repeated measures). Bonferroni test was used to adjust for multiple comparisons (SBP and heart rate). A *P* value of 0.05 or less was considered significant.

## Results

**Fig. 1:**
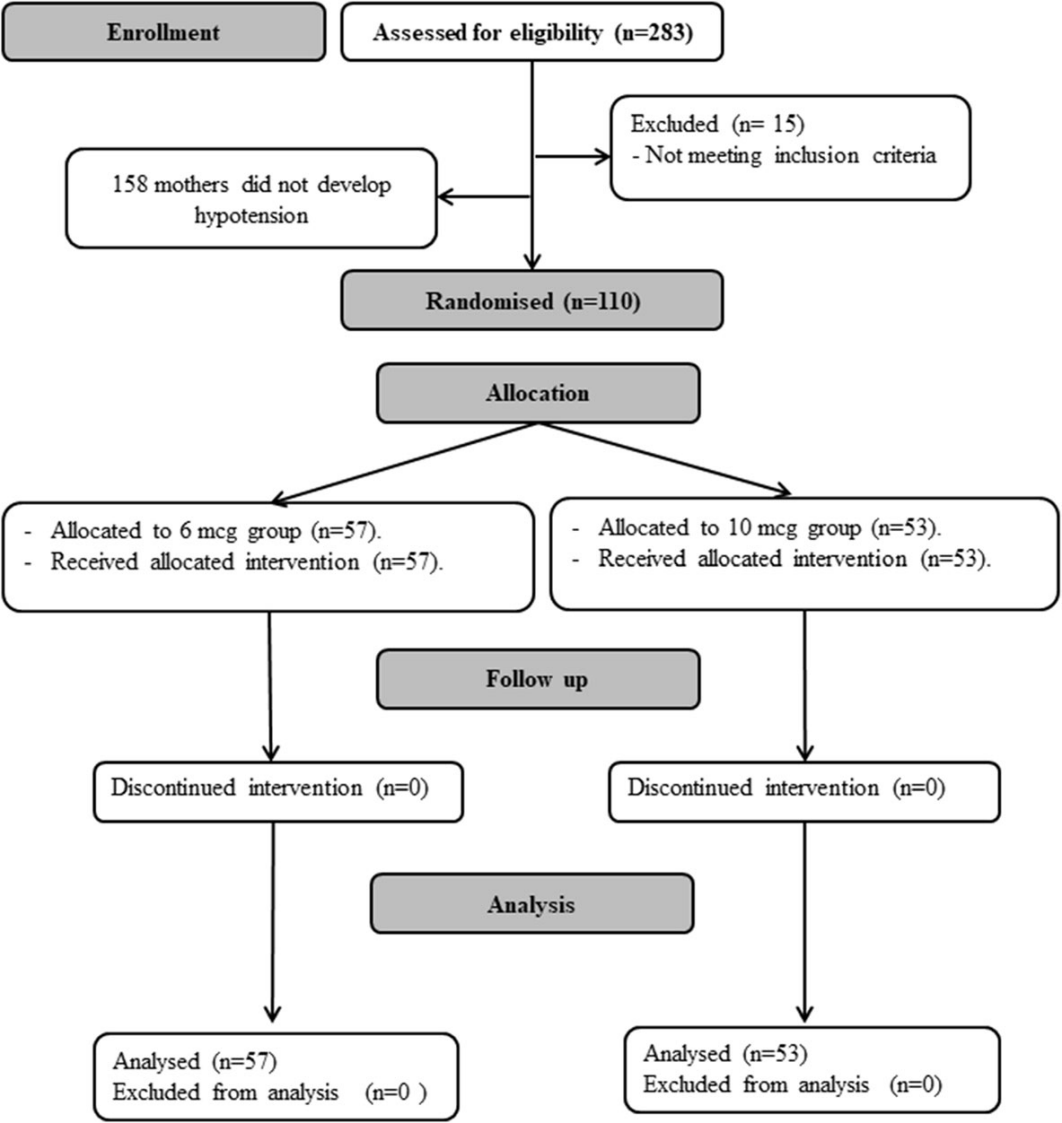
CONSORT chart showing patient recruitment

**Fig. 2:**
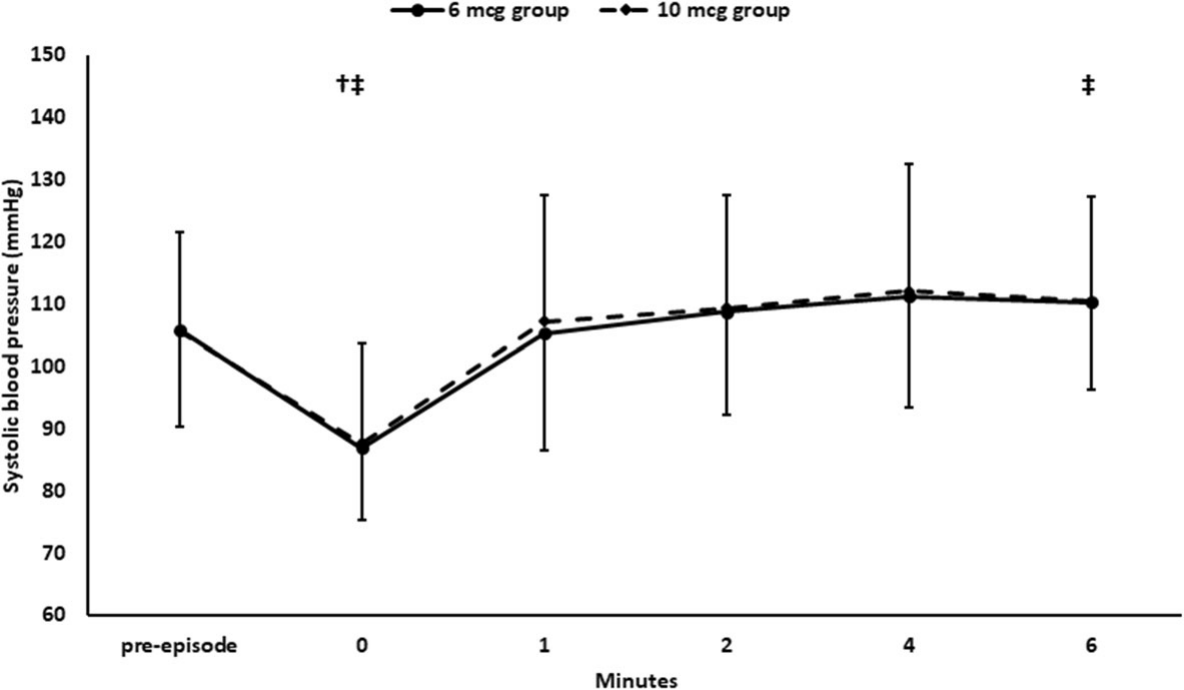
Systolic blood pressure. SBP: systolic blood pressure. Markers are means and error bars are standard deviations. *denotes statistical significance between both 6 mcg group and 10 mcg group. † denotes statistical significance compared to the pre-episodes reading within 6 mcg group, ‡ denotes statistical significance compared to the pre-episodes reading within 10 mcg group. Bonferroni test was used to adjust for multiple comparisons

**Fig. 3:**
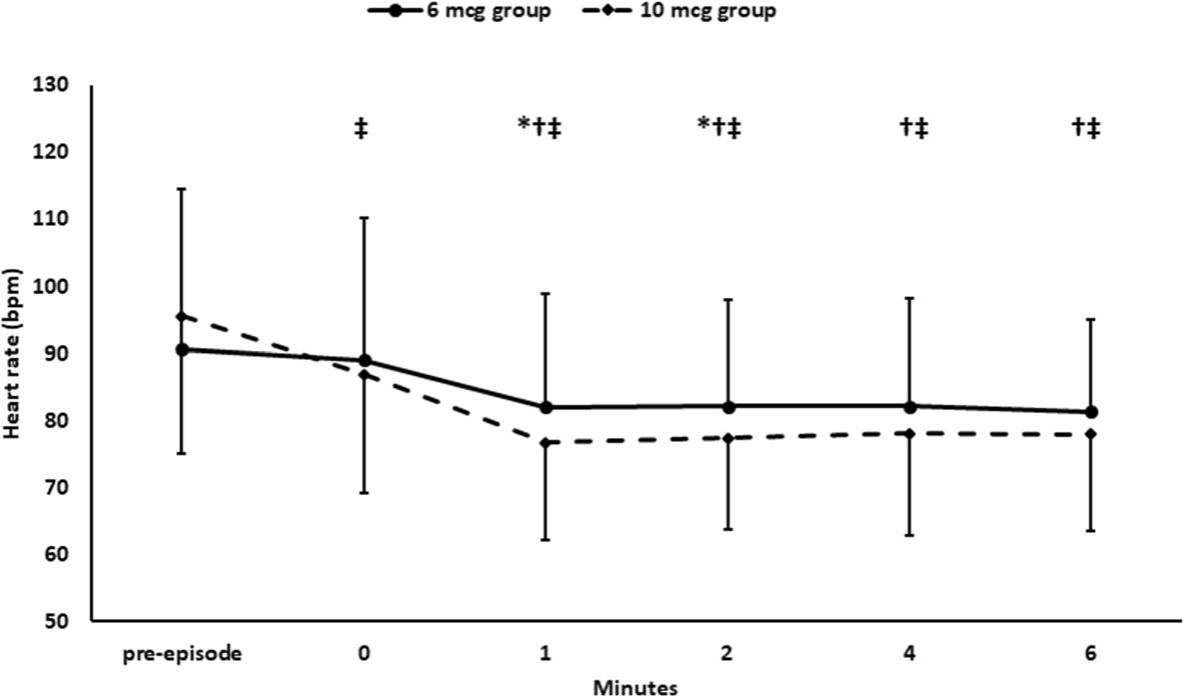
Heart rate. Markers are means and error bars are standard deviations. *denotes statistical significance between both 6 mcg group and 10 mcg group. † denotes statistical significance compared to the pre-episodes reading within 6 mcg group, ‡ denotes statistical significance compared to the pre-episodes reading within 10 mcg group. Bonferroni test was used to adjust for multiple comparisons

One hundred and ten mothers with post-spinal hypotension were included in the study. The patients had a total number of 110 first hypotensive episodes (Fig. 1). Baseline hemodynamic characteristics and demographic data were comparable between the two groups. (Table 1) Fifty-seven hypotensive episodes were treated by 6 mcg NE, with a success rate of 50/57 (88%), while 53 hypotensive episodes were treated by 10 mcg NE with a success rate of 45/53 (85%). Comparison of the 6 mcg-treated and the 10 mcg-treated episodes revealed a comparable rate of successful management and comparable SBP readings after the NE bolus (Table 2) (Fig. 2). The heart rate was lower in the 10 mcg-treated episodes (Fig. 3); however, we did not encounter any case of severe bradycardia requiring atropine injection after administration of either dose (Table 2).

**Table 1:** Baseline hemodynamic characteristics and demographic data. Data presented as mean (standard deviation) and median (quartiles)

	6 mcg group (*n* = 57)	10 mcg group (*n* = 53)	*P* value
Age (years)	28.6 (4.1)	29.9 (4.6)	0.15
Weight (Kg)	75 (70, 90)	80.5 (74.8, 90)	0.31
Time from spinal block to delivery (min)	18 (12, 26)	20 (12.5, 25.5)	0.26
Baseline systolic blood pressure (mmHg)	119.8 (9.7)	121.5 (9.8)	0.80
Baseline Heart rate (bpm)	96.8 (14.5)	97.2 (14.7)	0.74

**Table 2:** Characteristics of the hypotensive episodes. Data presented as frequency (%)

	6 mcg group (n = 57)	10 mcg group (n = 53)	*P* value
Successfully treated episode (%)	50/57 (88%)	45/53 (85%)	0.78
Reactive hypertension after NE bolus (%)	5/57 (9%)	5/53 (9%)	1.00
Severe hypotensive episodes (%)	11 (14.7)	15 (19.7)	0.52
Successfully treated severe episodes (%)	10/11 (90.9)	13/15 (86.7)	1.00
Reactive bradycardia after norepinephrine bolus (%)	5/57 (9%)	5/53 (9%)	1.00

Maternal outcomes, including number of hypotensive episodes, incidence of bradycardia, incidence of nausea and vomiting, total NE consumption, incidence of severe hypotensive episodes, and neonatal outcomes were not significantly different between the two groups (Table 3).

**Table 3:** Maternal outcomes and Neonatal outcomes. Data presented as median (quartiles) and frequency (%)

	6 mcg group (n = 57)	10 mcg group (n = 53)	*P* value
Maternal outcomes			
Number of hypotensive episodes per mother	1 (1,2)	1 (1,2)	0.23
Incidence of bradycardia (%)	4 (7%)	7 (13.2%)	0.35
Incidence of nausea and vomiting (%)	10 (17.5%)	5 (9.4%)	0.27
Total norepinephrine consumption (mcg)	124 (102, 152)	118.8 (95, 137.2)	0.40
Neonatal outcomes			
Apgar score at 1 min	7 (6, 9)	8 (6, 9)	0.44
Apgar score at 5 min	9 (9, 10)	9 (9, 10)	0.60
Umbilical artery *p*H	7.29 (7.21, 7.33)	7.31 (7.21, 7.35)	0.25
Umbilical artery PO2 (mmHg)	21 (12.7, 29)	24 (18, 27.3)	0.21
Umbilical artery PCO2 (mmHg)	48 (41, 59)	43.5 (37, 54.3)	0.07
Umbilical artery HCO3 (mmol. L^−1^)	22 (18.2, 24.3)	22 (18, 24)	0.79
Umbilical artery Lactate (mmol. L^−1^)	2.4 (1.5, 3.6)	2.7 (1.5, 5.1)	0.62

## Discussion

We compared two bolus doses of NE for management of post-spinal hypotension and found that the higher dose was not any superior to the lower dose. The success rate was ∼ 85% with both doses. The SBP was comparable after administration of either dose. The heart rate was modestly lower after administration of the 10 mcg bolus; however, no episodes of bradycardia needing atropine occurred after NE boluses administration.

The use of NE infusion for prophylaxis against post-spinal hypotension has demonstrated acceptable results. However, with most prophylactic regimens, there are some mothers who experience hypotension and need additional vasopressor boluses. The use of NE boluses for management of hypotension has not been adequately investigated. Some doses for NE boluses had been previously suggested. Onwochei et al. [9] had reported a 95% effective dose (ED95) of 5.8 mcg. Ngan Kee [10] had reported a 50% effective dose (ED50) of 10 mcg, and Mohta et al. had found that ED95 for NE bolus was 3.7 mcg [11]. Our study had a different design from the previous work as it aimed to manage hypotension in mothers who were already receiving prophylactic NE infusion. We selected our doses after revisiting the available data, in addition to our unpublished daily practice. We used a lower dose of 6 mcg; this dose was selected as the nearest dose to the 5.8 mcg dose which was used by Onwochei et al., for prophylaxis against maternal hypotension. We hypothesized that the dose needed for the management of hypotension might be higher than the dose needed for prophylaxis; therefore, we compared the 6 mcg dose with a higher dose of 10 mcg.

The higher dose in our study (10 mcg) was selected after revisiting Ngan Kee’s graded dose-response study [10], in which he reported that the ED50 for NE was 10 mcg with a 95% confidence interval between 6 mcg and 17 mcg. Our study showed that the effect of the 10 mcg bolus was not superior to that of the 6 mcg bolus. Our study differed from the Ngan Kee’s study [10] in the presence of background prophylactic vasopressor infusion in our patients. This might explain the high success rate of the low dose of NE bolus, namely 6 mcg, in our patients. Another important difference between our study and Ngan Kee’s study is the objective of both studies. The principal objective of Ngan Kee’s study [10] was to find the accurate relative potency between NE and phenylephrine; whilst, the objective of our study was to evaluate the efficacy of two bolus doses of NE in a large number of hypotensive episodes. Finally, Ngan Kee used a different endpoint than ours because he aimed to restore SBP to the baseline reading while our objective was to restore SBP to > 80% of the baseline reading.

In a recent dose-response study, which was not available when we started recruiting our patients, Mohta et al. found that the ED95 for NE bolus was 3.7 mcg which is lower than our doses [11]. However, Mohta et al. had a different experimental design compared to ours. Mohta et al. [11] recruited 50 mothers and started with a 6 mcg bolus, then performed an incremental reduction of the dose. Thus, only one mother among their participants received 6 mcg, 5.5 mcg, 5 mcg, or 4.5 mcg bolus doses each. Meanwhile, 20 patients received 4 mcg and 23 patients received 3.5 mcg. We suggest that adequate evaluation of success rate of the NE bolus dose requires inclusion of more hypotensive episodes. This suggestion is supported by the results of Ngan Kee’s study who reported a larger ED50 dose, 10 mcg [10]. Thus, we suggest that the study by Mohta et al. did not provide adequate evidence to support the use of a specific dose.

The incidence of maternal hypotension in our patients was 38%. This incidence is similar to the incidence which was reported by Wei et al. who used the same dose of NE infusion [12]. However, this incidence was relatively higher than that reported in our previous studies about cesarean delivery (∼ 30%) [5, 6, 13]. There is a variable incidence of hypotension among previous reports in which vasopressor prophylaxis was used during cesarean delivery; this incidence ranged between 2% [14] and 49% [15]. We also acknowledge that the incidence of hypotension in the current study was lower than that reported in our studies in which no vasopressor was used for prophylaxis (∼ 60%) [16, 17]. Therefore, we assume that using vasopressor prophylaxis would decrease the incidence of maternal hypotension compared to no-vasopressor protocols. However, there would be a variable incidence of hypotension, which should be necessarily treated using vasopressor boluses. This fact supports the need for studies that evaluate different bolus doses in treating hypotensive episodes. We used NE infusion at a dose of 0.05 mcg. Kg^− 1^.min^− 1^; this dose was previously reported by our group as a reasonable starting dose for NE during cesarean delivery [6]. Two recent studies had suggested higher doses (0.07 mcg. Kg^− 1^.min^− 1^ [12] and 0.08 mcg. Kg^− 1^.min^− 1^ [18]); however, these studies were not available when we started our study.

The findings of this study have several implications relevant for clinical management. 1. We report that there is no advantage in the use of 10 mcg NE bolus dose over 6 mcg NE bolus for management of maternal hypotension in the presence of NE infusion, even with the episodes of severe hypotension, the success rate was comparable in the two groups. 2. Neither of the two doses reached 100% success rate. 3. Neither of the two doses resulted in significant bradycardia, despite the lower heart rate readings after administration of the 10 mcg dose. Our study had the advantage of being the first study that evaluated the success rate of NE boluses in 110 hypotensive episodes. Moreover, it is the first study to evaluate the efficacy (success in management of hypotension), and the safety (the impact on heart rate) of NE boluses in mothers who are under prophylactic vasopressor infusion; therefore, we evaluated NE boluses using a study design which is adherent to the current guidelines.

The study had some limitations: 1. It is a single center study. 2. We did not include mothers with hypertensive disorders of pregnancy. 3. The number of severe hypotensive episodes was not enough to compare the two study doses. 4. We did not evaluate the repeated episodes. The.

current question that might need further research is: Considering that administration of a 10 mcg NE bolus was not associated with persistent bradycardia nor hypertension, could we try a higher dose of NE bolus for management of hypotension aiming for a 100% success rate?

## Conclusion

In mothers undergoing elective cesarean delivery under prophylactic NE infusion at 0.05 mcg. Kg^− 1^.min^− 1^, there was no advantage to the use of 10 mcg NE bolus over 6 mcg NE bolus for the rescue management of hypotensive episodes. Neither of the 2 bolus doses reached a 100% success rate. The incidences of bradycardia and reactive hypertension were comparable between both NE doses.

## Data Availability

The data that support the findings of this study are available from Cairo university hospitals; however, they are not publicly available. Data are however available from the corresponding author upon reasonable request after permission of Cairo university.

## Abbreviations

NE: Norepinephrine
SBP: Systolic blood pressure
SPSS: Statistical package for social science
ANOVA: Analysis of variance
ED: Effective dose
